# Supplementary material for: 1/f2 spectra of decoherence noise on 75As nuclear spins in bulk GaAs
Source: Sci Rep. 2020 Jun 30;10:10674. doi: 10.1038/s41598-020-67636-9 (PMC7326918; doi:10.1038/s41598-020-67636-9)
Supplement: Supplementary file 1 — Supplementary information [file 41598_2020_67636_MOESM1_ESM.pdf]

Supplementary Information to

$1/f^2$ spectra of decoherence noise on ^{75}As nuclear spins in bulk GaAs

*Susumu Sasaki[1,2], #Takanori Miura[1], #Kosuke Ikeda[1], #Masahiro Sakai[1],

+Takuya Sekikawa[3], +Masaki Saito[3], Tatsuro Yuge[4], Yoshiro Hirayama[5]

[1] Materials Science Program, Niigata University, Niigata 950-2181, Japan
[2]Japan Agency for Medical Research Development, Tokyo 100-0004, Japan
[3]Graduate School of Science and Technology, Niigata University, Niigata 950-2181, Japan
[4]Department of Physics, Shizuoka University, Shizuoka 422-8529, Japan
[5]Department of Physics, Tohoku University, Sendai 980-8578, Japan
#,+These authors contributed equally to this work.

[SI-1] Figure shows ^{75}As-NMR frequency spectra (a) un-doped, (b) Cr-doped semi-insulating and (c) Si-doped metallic GaAs substrate. Spin-echo intensities are plotted with varying resonance frequency point-by-point. By minimising the RF power, we realised the frequency resolution of (a)1.8 kHz, (b) 1.9 kHz, and (c) 1.9 kHz. The spectra are well-fit by a single Gaussian for both (a) and (b), wheres by a single Lorentian for (c). Full-width at half-maximum (FWHM) are (a)5.8 kHz, (b)6.4 kHz, and (c) 6.8 kHz.

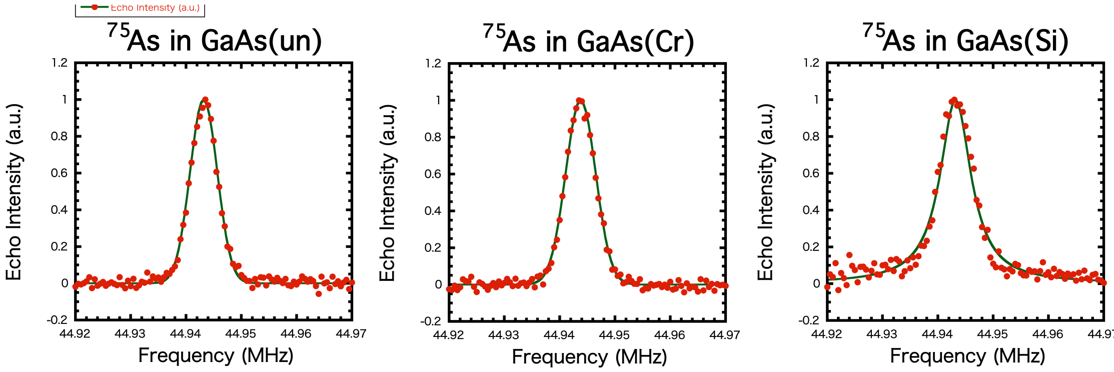

[SI-2] The Hahn-echo decay of ^{75}As for (a) un-doped, (b) Cr-doped semi-insulating and (c) Si-doped metallic GaAs substrate. All the decays are decomposed into a Gaussian function dominant in the initial process characterised by the time constant T_{2G} and a Lorentzian decay emergent in the long-time limit denoted by T_{2L}. Although T_2^L is also defined by the decay in the long-time limit, it is the time constant valid only in the presence of multiple π-pulses.

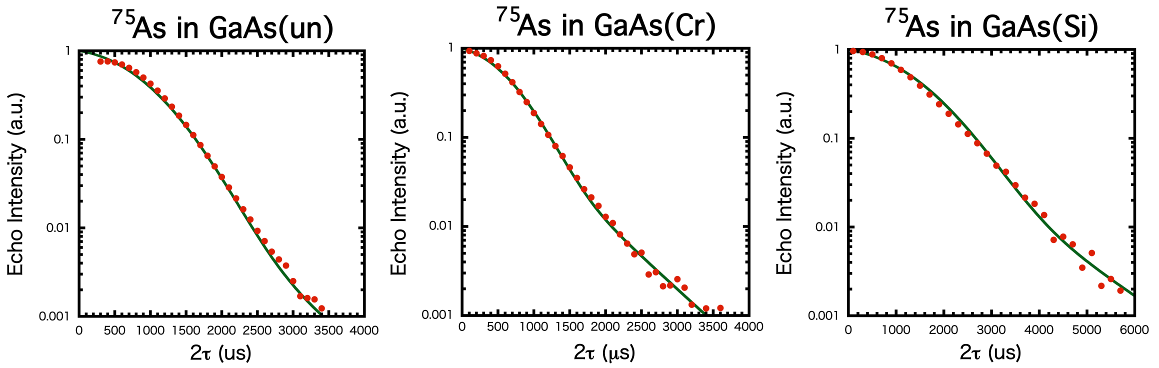

[SI-3] To deduce the slope in the long-time limit, or equivalently, to eliminate the extrinsic initial slopes, we first employ a two-component fitting as

$$s_{\mathrm{APCP}}(t) = F_{\mathrm{S}}\exp(-t/T_2^{\mathrm{S}}) + F_{\mathrm{L}}\exp(-t/T_2^{\mathrm{L}}) \qquad \text{(S1)}$$

with $F_{\mathrm{S}} + F_{\mathrm{L}} = 1$. **Left:** For shorter ($2\tau = 30$ μs) and longer ($2\tau = 600$ μs) pulse intervals, the data are well-fit by Eq. S1. **Right:** For intermediate pulse intervals ($2\tau = 300$ μs), the T_2^{L} in Eq. (S1) results in smaller values than the real final slope.

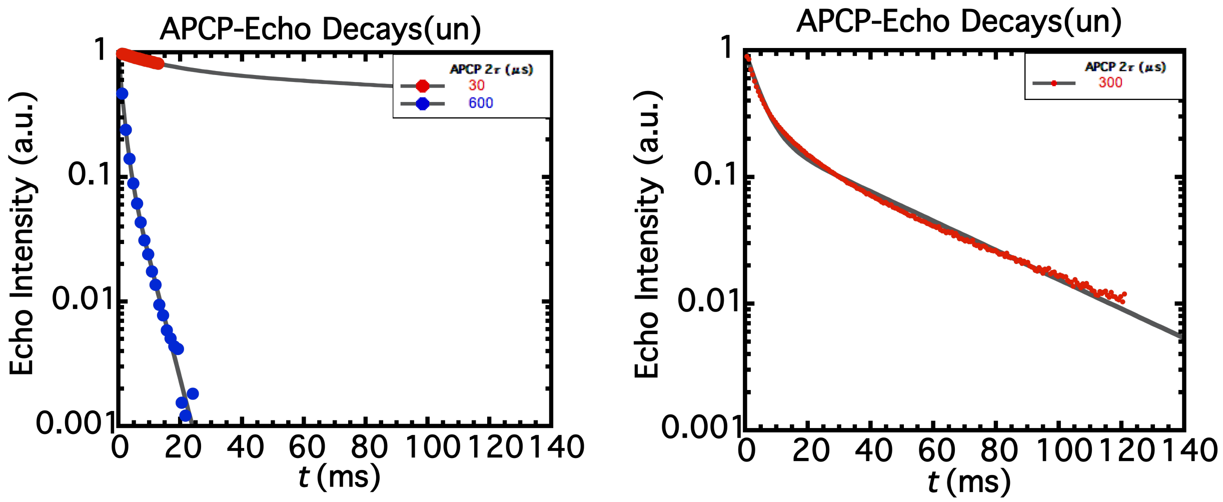

[SI-4] As a result of the failure of fitting by Eq. (S1), we employ a three-component fitting as

$$s_{\mathrm{APCP}}(t) = F_{\mathrm{S}}\exp(-t/T_2^{\mathrm{S}}) + F_{\mathrm{M}}\exp(-t/T_2^{\mathrm{M}}) + F_{\mathrm{L}}\exp(-t/T_2^{\mathrm{L}}) \qquad \text{(S2)}$$

with $F_{\mathrm{S}} + F_{\mathrm{M}} + F_{\mathrm{L}} = 1$. It is found that Eq. (S2) reproduces all the decay data with the right value of the slope in the long-time limit. Figure shows fraction F_{S}, F_{M} and F_{L} in Eq. (S2) dependences on pulse intervals 4τ for (a) un-doped, (b) Cr-doped semi-insulating and (c) Si-doped metallic GaAs. The gradual decrease in F_{L} with the increase in 4τ proves that the T_2^{L} values obtained by (S2) are intrinsic.

	un-doped	Cr-doped	Si-doped
$90PW_{APCP}$ (μs)	2.2	8	8
Δf_{APCP} (kHz)	114	31.3	31.3
Δf_{PBP} (kHz)	1.8	1.9	1.9
FWHM (kHz)	5.8	6.4	6.8
fc (Hz)	170 ± 10 (5.9%)	210 ± 10 (4.8%)	460 ± 30 (6.5%)
A term (Hz)	1.6 ± 0.05 (3.1%)	1.7 ± 0.08 (4.7%)	0.85 ± 0.04 (4.7%)
B term (Hz)	7.3 ± 0.4 (5.5%)	24.7 ± 0.9 (3.6%)	15.5 ± 0.7 (4.5%)
$T_2^{Hahn\_L}$ (ms)	0.64	0.59	1.18
T_1 (ms)	239.39	242.99	230.19
$T_2^{Hahn\_L}/T_1$ (%)	0.26	0.24	0.51

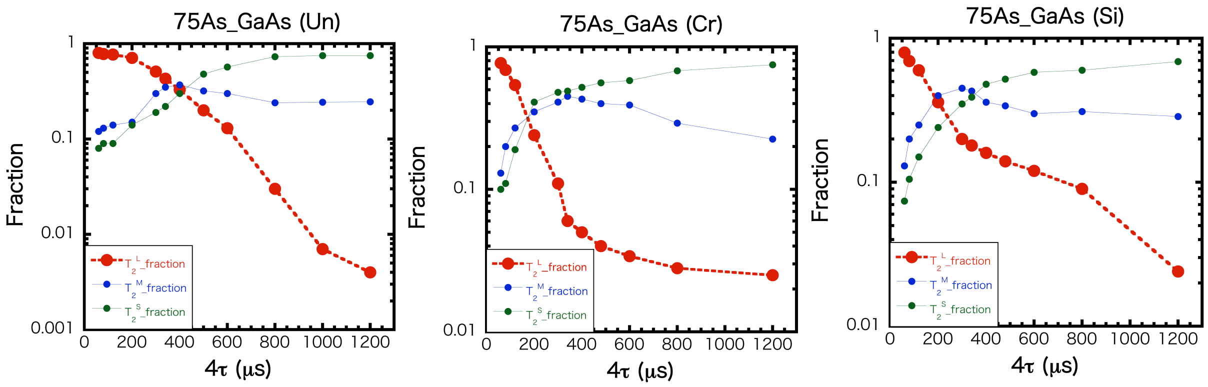

[SI-5] We summarize the values obtained throughout the present experiment in the following table.
